# The effect of sociodemographic, socioeconomic, and health factors on healthcare utilization in cardiovascular patients in Serbia: a part of National Health Survey

**DOI:** 10.3389/fpubh.2025.1569741

**Published:** 2025-07-17

**Authors:** Tamara Lekovic, Nikoleta Janicijevic, Milos Potezica, Nela Djonovic, Dragan Vasiljevic, Katarina Janicijevic, Melanija Tepavcevic, Snezana Knezevic, Jelena Vuckovic Filipovic, Amela Rastoder Celebic, Svetlana Vukosavljevic, Maja Mirocevic Rotolo, Dalibor Stajic

**Affiliations:** ^1^Faculty of Medical Sciences, University of Kragujevac, Kragujevac, Serbia; ^2^Department of Hygiene and Ecology, Institute of Public Health Kragujevac, Kragujevac, Serbia; ^3^Department of Hygiene and Ecology, Faculty of Medical Sciences, University of Kragujevac, Kragujevac, Serbia; ^4^Department of Social Medicine, Faculty of Medical Sciences, University of Kragujevac, Kragujevac, Serbia; ^5^Department of Anatomy, Faculty of Medical Sciences, University of Kragujevac, Kragujevac, Serbia; ^6^Department of Medical Sciences, Academy of Applied Studies Polytechnic, Belgrade, Serbia; ^7^Clinic for Cardiology, University Clinical Center Kragujevac, Kragujevac, Serbia; ^8^Department of Internal Medicine, Faculty of Medical Sciences, University of Kragujevac, Kragujevac, Serbia; ^9^Health Center Niksic, Niksic, Montenegro; ^10^Health Center Dimitrije Pitovic, Kosjeric, Serbia; ^11^A3 Medical Montenegro, Sutomore, Montenegro

**Keywords:** cardiovascular diseases, healthcare utilization, National Health Survey, sociodemographic factors, socioeconomic factors, Serbia

## Abstract

**Objectives:**

This cross-sectional analytical study explores the predictors of the healthcare utilization in the adult population with cardiovascular diseases.

**Methodology:**

The research was carried out as part of the fourth Serbian National Health Survey, in the period from October to December 2019, as a descriptive, cross-sectional analytical study and included the population of cardiovascular patients (*N* = 4,712) aged over 20 years; Descriptive and inferential statistical methods, including percentage-based structure indicators, Chi-square (χ2) tests for categorical variable differences, and prevalence ratio for examining relationships between dependent and independent variables, were used in the data analysis.

**Results:**

The analysis identified several significant contributors to cardiovascular healthcare utilization, emphasizing distinct and overlapping factors that impact various types of healthcare use. The chi-square test had shown that predominantly, each form of healthcare utilization was most prevalent among the 60–69 age group (general practitioner visits: 33.9%, specialist visits: 33.1%, hospitalization: 31.4%, *p* < 0.001). Self-assessment of general health (bad/very bad) and the presence of multiple chronic conditions (multimorbidity) were positively associated with general practitioner (PR = 1.037(1.001–1.075); *p* = 0.044, PR = 1.077(1.039–1.117); *p* < 0.001), specialist visits (PR = 1.381(1.281–1.490); *p* < 0.001, PR = 1.279(1.181–1.385); *p* < 0.001) and hospitalization (PR = 4.346(3.272–5.772); *p* < 0.001, PR = 2.018(1.517–2.685); *p* < 0.001).

**Conclusion:**

Sociodemographic, socioeconomic factors and some aspects of health status have a significant impact on the cardiovascular healthcare, thereby precise recommendations for facilitating appropriate healthcare utilization should be established. A top priority for academics, physicians and public health specialists is to keep emphasizing the significance of sociodemographic determinants in lowering cardiovascular complications, as well as strengthening cardiovascular healthcare.

## 1 Introduction

At the global level, cardiovascular diseases (CVD) are reaching epidemic proportions, especially in developing countries. They are the primary cause of morbidity and mortality worldwide, with heart attacks and strokes accounting for approximately 85% of these deaths. More than a third of deaths globally are attributed to cardiovascular diseases, which are thought to claim 17.9 million lives annually (1, 2). Regarding the trends in cardiovascular mortality rate in Serbia, data from 2007 indicated that the number of deaths was 57,608 (3), in 2014 it was 53,993 (4) and during 2021, 56.610 people died, which demonstrates that cardiovascular diseases are responsible for nearly half of all deaths each year (5). Previous National Health Surveys have highlighted a notable rise in the prevalence of cardiovascular diseases, with hypertension emerging as the predominant condition compared to all of the other diseases (19.7% in 2000, 23.1% in 2006, and 33.2% in 2013). This is followed by myocardial infarction (3.3% in 2006 and 2.6% in 2013) and stroke (2.0% in 2000, 2.7% in 2006, and 3.6% in 2013) (6). It can be concluded that cardiovascular diseases have a considerable impact, particularly in low- and middle-income countries (2).

The Republic of Serbia maintains a longstanding tradition of a publicly funded healthcare system, which operates under an obligatory social health insurance model. This system is financed through salary contributions made by employees and managed by the Health Insurance Fund (HIF). Budget allocations to the HIF ensure that health insurance coverage is extended to unemployed individuals, internally displaced persons, refugees, and others in vulnerable groups (7). Retired individuals are, as well, eligible for health insurance based on the contributions made during their employment (8). Due to the lack of private health insurance, private funding for healthcare is predominantly reliant on out-of-pocket payments (7).

In cardiovascular patients, primary and specialized care in Serbia are closely integrated through a collaborative approach, ensuring continuous and comprehensive management. General practitioners are essential in the early identification, prevention, and long-term care of cardiovascular patients. When advanced diagnostics or procedures are required, they also act as gatekeepers, referring patients to specialists. Cardiologists and other specialists provide targeted treatment and expert care for complex or acute conditions. Once stabilized, patients often return to their General practitioners for follow-up and ongoing monitoring. This coordinated relationship enhances patient outcomes, ensures continuity of care, and optimizes resource use by reducing unnecessary specialist visits (6). According to the Health Statistical Yearbook of the Republic of Serbia 2023, Serbia has 0.64 general practitioners and 2.01 specialists per 1,000 inhabitants (9). In summary, although Serbia may have a sufficient number of specialists, the low number of general practitioners may result in challenges in providing timely and comprehensive healthcare, particularly in rural or underserved areas. Increasing the number of general practitioners and ensuring better geographic distribution of healthcare providers would likely be beneficial for improving overall healthcare access and quality. Healthcare utilization is considered an important public health issue and is affected by various factors, including biological, environmental, and sociodemographic differences—such as gender, geographic location, educational access and income levels—that contribute to disparities among population groups and necessitate attention (10, 11).

Whereas the majority of research assesses the risk of cardiovascular disease occurrence, few investigate the role of factors influencing cardiovascular healthcare utilization (12). Previous research has identified several factors linked to higher healthcare utilization, such as living alone, poor self-rated health, having multiple chronic conditions, higher education levels, advanced age and higher income (13). The Prospective Urban Rural Epidemiologic (PURE) study highlights that individuals with lower socioeconomic status, especially those with limited access to education and income, are less likely to seek healthcare services consistently or on a routine basis. This lack of access is linked to a higher risk of cardiovascular diseases due to inadequate prevention, diagnosis, and treatment (14). Another research in low- and middle-income countries highlights that CVD healthcare utilization is severely limited due to poor access to care and poverty, which contributes to delays in diagnosis and treatment, exacerbating health disparities and worsen the burden of CVD in these regions (15).

Cardiovascular health components, including conditions such as obesity and high blood lipids are closely linked to healthcare utilization, making prevention of these modifiable factors vital for cardiovascular healthcare (16–18). A study in Greece concluded that individuals with higher body mass index (BMI) levels tend to have more physician consultations, particularly for cardiovascular conditions (19). A cross-sectional observational study conducted in 18 countries in Eastern Europe, Asia, Africa, the Middle East and Latin America, evaluated that patients with high blood lipids, particularly those at high cardiovascular risk, tend to have higher healthcare consumption, including more frequent visits to healthcare providers (18). A study assessing cardiovascular health in Serbia found that, unfortunately, only the minority of the population had satisfies the requirements for optimal cardiovascular health (20).

All of the aforementioned points highlight the significance of this study and the lack of research in Serbia and other similar countries on the impact of sociodemographic, socioeconomic and health factors on cardiovascular healthcare. Therefore the further analysis is important for the health system in Serbia and Balkan, in order to identify factors that may have an impact on the occurrence of consequences, which may further worsen the health condition of cardiovascular patients. Such research can contribute to the formulation of health strategies and policies, which would improve health, reduce the incidence of serious consequences of cardiovascular diseases and prevent/delay potential death, and consequently improve health.

## 2 Methodology

### 2.1 Study type, population, sampling

This study utilized data from the 2019 Serbian National Health Survey, which is a cross-sectional nationwide survey conducted by the Institute of Statistics of the Republic of Serbia in collaboration with the Institute of Public Health “Dr. Milan Jovanović Batut” and the Ministry of Health (21). Survey target population covers non-institutionalized individuals aged 15 or more years old, residents of Serbia. The survey sample (*N* = 13,178) was selected using a two-stage stratified probability-based cluster design to be representative at the national level as well as at the level of four regions (Belgrade region, Vojvodina region, Sumadija and Western Serbia region, Southern and Eastern Serbia region), following methodological guidelines of the European Health Survey—third wave (EHIS-wave 3) (Supplementary data 2) (21). The study applied a stratified two-stage cluster sampling method. In the first stage, a random sample of census districts (household groups) was selected with a probability proportional to their size. In the second stage, households within each census district were selected with equal probability. The population census conducted in the Republic of Serbia in 2011 was used as the sampling frame. For this study, we considered participants aged 20 years or above with at least one of the following reported health conditions: myocardial infarction, angina pectoris, hypertension and stroke (*n* = 4,712) (Supplementary data 1). The data was collected from October to December 2019 by the Institute of Statistics of the Republic of Serbia in cooperation with the Institute of Public Health of Serbia „Dr. Milan Jovanović Batut “and the Ministry of Health of the Republic of Serbia, using a standardised EHIS 3 questionnaire (21).

### 2.2 Research instruments

A standardized questionnaire (European Health Interview Survey—EHIS) was used as the main research instrument. The questionnaire in question was used in various health population surveys in several countries of the European Union and was adapted to the cultural characteristics of our area.

Two types of questionnaires were used in the research:

1. Information panel for the household, which is used to collect information about all members of the household, i.e., about the socio-economic characteristics of the household itself.
2. Questionnaire filled out for each household member aged 15 and over [sociodemographic, socioeconomic characteristics and health factors: sex, age, region, marital status, education, employment status, well-being index, self-assessment of general health, chronic diseases, high blood lipids, and utilization of healthcare services: general practitioner visits, specialists visits and hospitalizations in the preceding twelve months, basic anthropogenic measurements (measured by the examiner): height, weight and blood pressure] (21).

The body mass index (BMI) is a height-weight indicator of an individual’s nutrition and is calculated by dividing the person’s body mass in kilograms by the square of the height in meters (below 18.5—malnutrition, 18.5–24.9—normal nutrition, 25.0–29.9—overweight, 30.0 and above—obesity) (22).

### 2.3 Measurement instruments

Sociodemographic, socioeconomic, and health aspects were gathered through standardized face-to-face interviews conducted in participants’ households. The independent variables used in the research are: sociodemographic, socioeconomic and health factors (gender: male/female, age: 20–29/30–39/40–49/50–59/60–69/70–79/80+, region: capital/northern/central and western/south and eastern, marital status: never married/divorced/married, education: primary/secondary/advanced, employment status: unemployed/inactive/employed, well-being index: poorest/middle class/wealthiest, self-assessment of general health: bad, very bad/average/good, very good, chronic diseases: multimorbidity/1 disease, high blood lipids: yes/no and BMI: malnutrition/normal/pre-obesity/obesity class I/ obesity class II/ obesity class III) while the dependent variable was the cardiovascular healthcare utilization (general practitioner visits, specialist visits and hospitalization).

### 2.4 Ethical and legal aspects

The international Declaration of Helsinki complied with ethical standards and there were no deviations from the principles of scientific research work.

The respondents voluntarily gave their consent to participate in the research, by signing the informed consent. The collection of data that identifies the respondent was also avoided (necessary identifiers were replaced by a code), and the results were published in such a way that the confidentiality of individual data was fully ensured (21).

### 2.5 Statistical methods

The statistical program SPSS version 22 was used to analyse and display the data.

In statistical data processing, descriptive and inferential statistical methods were used in data analysis. The data were of a categorical type, so the structure indicators, expressed as percentages were used to describe the data. Chi-square (χ2) was used to compare differences in the frequency of categorical variables. Testing the difference in the distribution of two or more observation features was performed with the χ2 test in the form of rxk-type contingency tables.

The relationship between dependent variables and a series of independent variables was examined by prevalence ratio. Prevalence ratios (PR) were estimated using Poisson regression models with robust variance estimation to directly calculate adjusted PRs. This approach was chosen to provide interpretable measures of association for common outcomes, avoiding the overestimation inherent to odds ratios in such contexts. Both univariable and multivariable Poisson regression models were conducted to examine the relationship between independent variables and the outcome. Adjusted PRs with 95% confidence intervals (CIs) were reported. Statistical significance was set at *p* < 0.05.

## 3 Results

The study included 4,712 participants, out of whom 2,624 (55.7%) were female and 2088 (44.3%) were male respondents. The largest percentage of participants (32.6%) were aged between 60–69, predominately resided in the central and western region (30.1%) and gave an affirmative answer to the question about marital status in terms of living in a union (65.5%) and in terms of educational structure, secondary education was the most common (48.2%). The general impression, obtained by processing the collected data, was that the surveyed population had mostly inactive employment status (67.6%) and the analysis revealed that most individuals belonged to the poorest segment of society. Through analysis, we concluded that in the cardiovascular population, the self-assessment of general health was predominantly rated as average. This group exhibited a higher prevalence of chronic diseases, pre-obesity, but did not show a significant presence of high blood lipids. When it comes to the healthcare utilization, the majority of the population suffering from cardiovascular diseases testified that they had not been hospitalized in the previous 12 months (86.4%), however, they had visited specialists (59.0%) and general practitioners (85.8%). Through analyzing general practitioner visits, we concluded that cardiovascular patients were predominantly in the 60–69 age group (*p* < 0.001), resided in the central and western region (*p* = 0.019), were inactive in terms of employment status (*p* < 0.001), rated their general health as average (*p* < 0.001), had more than 1 chronic disease (*p* < 0.001) and did not have elevated blood lipids (*p* < 0.001). The group that visited specialists exhibited identical significant differences to the population that visited general practitioners, with the addition of the well-being index being predominant in the poorest segment (*p* = 0.003). Regarding hospitalization, the following factors were identified as significant: gender (*p* = 0.004), age (*p* < 0.001), marital status (*p* = 0.027), education (*p* = 0.002), employment status (*p* < 0.001), well-being index (*p* = 0.023), self-assessment of general health, chronic disease (*p* < 0.001), and high blood lipids (*p* = 0.014) (Table 1).

**Table 1:** Sociodemographic characteristics, socioeconomic characteristics, and health aspects of the respondents.

Variables	General practitioner visits	X^2^	Specialist visits	χ^2^	Hospitalization	X^2^	Study population *N* (%)
	*N* (%)		*N* (%)		*N* (%)		
Sex							
Female	2,256 (56.1)	χ2 = 2.228 *p* = 0.074	1,525 (55.6)	χ2 = 0.107 *p* = 0.383	325 (50.8)	χ2 = 11.103 ***p* = 0.004**	2,624 (55.7)
Male	1765 (43.9)		1,218 (44.4)		315 (49.2)		2088 (44.3)
Age							
20–29	37 (0.9)	χ2 = 75.846 ***p* < 0.001**	25 (0.9)	χ2 = 34.377 ***p* < 0.001**	6 (0.9)	χ2 = 34.957 ***p* < 0.001**	46 (1)
30–39	106 (2.6)		67 (2.4)		15 (2.3)		142 (3)
40–49	304 (7.6)		210 (7.7)		29 (4.4)		391 (8.3)
50–59	722 (18.0)		495 (18.0)		105 (16.3)		871 (18.5)
60–69	1,362 (33.9)		907 (33.1)		201 (31.4)		1,537 (32.6)
70–79	1,025 (25.5)		734 (26.8)		196 (30.5)		1,148 (24.3)
80+	465 (11.6)		305 (11.1)		91 (14.2)		577 (12.2)
Region							
Capital	818 (20.3)	χ2 = 9.949 ***p* = 0.019**	608 (22.2)	χ2 = 16.044 ***p* < 0.001**	141 (22.1)	χ2 = 7.031 *p* = 0.318	984 (20.9)
Northern	896 (22.3)		630 (23.0)		139 (21.7)		1,060 (22.5)
Central and Western Serbia	1,227 (30.5)		763 (27.8)		180 (28.1)		1,418 (30.1)
Southern and Eastern Serbia	1,080 (26.9)		742 (27.1)		180 (28.1)		1,250 (26.5)
Marital Status							
Never married, extramarital union	175 (4.3)	χ2 = 3.600 *p* = 0.165	121 (4.4)	χ2 = 3.949 *p* = 0.139	34 (5.2)	χ2 = 10.975 ***p* = 0.027**	225 (4.7)
Divorce, separation, death of a partner	1,199 (29.9)		788 (28.7)		213 (33.2)		1,403 (29.8)
Marriage, extramarital union	2,641 (65.8)		1833 (66.8)		394 (61.6)		3,084 (65.5)
Education							
Primary	1,486 (37.0)	χ2 = 4,945 *p* = 0,084	949 (34.6)	χ2 = 37.737 ***p* < 0.001**	275 (43.1)	χ2 = 14.458 ***p* = 0.002**	1776 (37.7)
Secondary	1957 (48.7)		1,346 (49.1)		297 (46.2)		2,271 (48.2)
Advanced	576 (14.3)		447 (16.3)		69 (10.7)		665 (14.1)
Employment Status							
Unemployed	465 (11.6)	χ2 = 46.775 ***p* < 0.001**	304 (11.1)	χ2 = 20.482 ***p* < 0.001**	82 (12.8)	χ2 = 36.655 ***p* < 0.001**	574 (12.2)
Inactive	2,792 (69.9)		1929 (70.6)		483 (76.0)		3,184 (67.6)
Employed	738 (18.5)		500 (18.3)		72 (11.2)		954 (20.2)
Well-being index							
Poorest	1752 (43.5)	χ2 = 2.365 *p* = 0.307	1,152 (42.0)	χ2 = 11.485 ***p* = 0.003**	310 (48.4)	χ2 = 11.362 ***p* = 0.023**	2078 (44.1)
Middle class	863 (21.5)		612 (22.3)		125 (19.5)		1,013 (21.5)
Wealthiest	1,406 (35.0)		979 (35.7)		208 (32.1)		1,621 (34.4)
Self-assessment of general health							
Bad/very bad	985 (25.4)	χ2 = 20.337 ***p* < 0.001**	762 (28.9)	χ2 = 119.28 ***p* < 0.001**	301 (50.0)	χ2 = 270.98 ***p* < 0.001**	1,180 (25)
Average	1,653 (42.6)		1,155 (43.9)		222 (37.0)		1964 (41.7)
Good/very good	1,240 (32.0)		718 (27.2)		80 (13.0)		1,568 (33.3)
Chronic diseases							
Multimorbidity	3,036 (75.5)	χ2 = 70.969 ***p* < 0.001**	2,200 (80.2)	χ2 = 156.561 ***p* < 0.001**	571 (88.9)	χ2 = 92.986 ***p* < 0.001**	3,450 (73.2)
1 disease	985 (24.5)		543 (19.8)		71 (11.1)		1,262 (26.8)
High blood lipids							
Yes	1,044 (26.1)	χ2 = 49.258 ***p* < 0.001**	803 (29.4)	χ2 = 93.127 ***p* < 0.001**	188 (29.7)	χ2 = 12.447 ***p* = 0.014**	1,140 (24.2)
No	2,952 (73.9)		1925 (70.6)		445 (70.3)		3,572 (75.8)
BMI							
Malnutrition	35 (1.0)	χ2 = 5.515 *p* = 0.356	22 (1.0)	χ2 = 4.806 *p* = 0.440	7 (1.4)	χ2 = 10.156 *p* = 0.427	47 (1)
Normal	844 (24.9)		556 (24.1)		130 (25.4)		1,193 (25.3)
Pre-obesity	1,360 (40.1)		942 (40.9)		202 (39.5)		1889 (40.1)
Class I	824 (24.3)		565 (24.5)		133 (26.0)		1,125 (23.9)
Class II	252 (7.4)		162 (7.0)		24 (4.7)		345 (7.3)
Class III	77 (2.3)		58 (2.5)		16 (3.1)		113 (2.4)

*Statistically significant results (*p* < 0.05) are presented in bold.

Univariable and multivariable prevalence ratio models were employed to analyse the statistically significant predictors of different types of healthcare utilization and included gender, age, region, marital status, education, employment status, well-being index, self-reported health, chronic diseases, high blood lipids and BMI. A visit to a general practitioner was significantly predicted by age (60–69: PR = 1.100(1.054–1.149); *p* < 0.001, 70–79: PR = 1.107(1.059–1.157); *p* < 0.001), region (capital: PR = 0.962(0.929–0.997); *p* = 0.032), employment status (Inactive: PR = 1.097(1.060–1.136); *p* < 0.001), self-assessment of health (bad/very bad: PR = 1.068(1.035–1.102); *p* < 0.001, average: PR = 1.052(1.022–1.083); *p* = 0.001), chronic diseases (multimorbidity: PR = 1.123(1.089–1.159); *p* < 0.001) and high blood lipids (yes: PR = 1.093(1.069–1.118); *p* < 0.001) in the univariate analysis. The multivariable analysis indicated that the statistically significant predictors were age, region, education, employment, self-assessment of general health, chronic diseases and high blood lipids (Table 2).

**Table 2:** Prevalence ratio analysis: association between sociodemographic characteristics, socioeconomic characteristics, health aspects, and general practitioner visits.

Variables	General practitioner visits			
	Univariable model		Multivariable model	
	PR (95%CI)	*p*	PR (95%CI)	*p*
Sex				
Female	1.018 (0.994–1.042)	0.138	1.013 (0.9861.040)	0.348
Male	1		1	
Age				
20–29	0.993 (0.856–1.151)	0.925	1.024 (0.866–1.212)	0.781
30–39	0.928 (0.837–1.028)	0.154	0.955 (0.848–1.076)	0.450
40–49	0.965 (0.903–1.030)	0.284	0.976 (0.900–1.059)	0.561
50–59	1.029 (0.979–1.081)	0.256	1.036 (0.976–1.099)	0.242
60–69	1.100 (1.054–1.149)	**<0.001**	1.055 (1.006–1.106)	**0.027**
70–79	1.107 (1.059–1.157)	**<0.001**	1.059 (1.011–1.110)	**0.016**
80+	1		1	
Region				
Capital	0.962 (0.929–0.997)	**0.032**	0.929 (0.890–0.970)	**0.001**
Northern	0.977 (0.945–1.010)	0.176	0.959 (0.926–0.994)	**0.021**
Central and Western Serbia	1.010 (0.980–1.040)	0.520	1.004 (0.974–1.036)	0.773
Southern and Eastern Serbia	1		1	
Marital status				
Never married, extramarital union	0.947 (0.887–1.011)	0.104	1.015 (0.945–1.091)	0.681
Divorce, separation, death of a partner	1.002 (0.977–1.028)	0.863	1.001 (0.973–1.030)	0.942
Marriage, extramarital union	1		1	
Education				
Primary	0.969 (0.935–1.004)	0.086	0.928 (0.890–0.967)	**<0.001**
Secondary	0.995 (0.962–1.029)	0.760	0.989 (0.954–1.026)	0.568
Advanced	1		1	
Employment status				
Unemployed	1.011 (0.961–1.064)	0.666	0.978 (0.924–1.034)	0.434
Inactive	1.097 (1.060–1.136)	**<0.001**	1.052 (1.002–1.105)	**0.043**
Employed	1		1	
Well-being index				
Poorest	0.979 (0.954–1.006)	0.121	0.987 (0.956–1.019)	0.413
Middle class	0.988 (0.958–1.020)	0.469	0.990 (0.957–1.025)	0.574
Wealthiest	1		1	
Self-assessment of general health				
Bad/very bad	1.068 (1.035–1.102)	**<0.001**	1.037 (1.001–1.075)	**0.044**
Average	1.052 (1.022–1.083)	**0.001**	1.019 (0.986–1.052)	0.267
Good/very good	1		1	
Chronic diseases				
Multimorbidity	1.123 (1.089–1.159)	**<0.001**	1.077 (1.039–1.117)	**<0.001**
1 disease	1		1	
High blood lipids				
Yes	1.123 (1.089–1.159)	**<0.001**	1.048 (1.022–1.075)	**<0.001**
No	1		1	
BMI				
Malnutrition	1.068 (0.745–0.901)	0.391	1.073 (0.922–1.249)	0.364
Normal	1.034 (0.919–1.242)	0.500	1.041 (0.944–1.147)	0.421
Pre-obesity	1.056 (0.959–1.164)	0.270	1.059 (0.962–1.166)	0.240
Obesity class I	1.070 (0.970–1.180)	0.175	1.076 (0.977–1.186)	0.138
Obesity class II	1.068 (0.962–1.186)	0.216	1.071 (0.966–1.187)	0.191
Obesity class III	1		1	

*Statistically significant results (*p* < 0.05) are presented in bold.

Based on both univariable and multivariable analyses, region (Central and Western Serbia: PR = 0.920(0.861–0.982); *p* = 0.013, PR = 0.919(0.859–0.983); *p* = 0.014), education (primary: PR = 0.800(0.748–0.856); *p* < 0.001, PR = 0.755(0.697–0.818); *p* < 0.001, secondary: PR = 0.879(0.826–0.936); *p* < 0.001, PR = 0.885(0.826–0.948); *p* = 0.001), well-being index (poorest: PR = 0.919(0.870–0.970); *p* = 0.002, PR = 0.933(0.872–0.997); *p* = 0.042), self-assessment of health (bad/very bad: PR = 1.426(1.335–1.523); *p* < 0.001, PR = 1.381(1.281–1.490); *p* < 0.001, average: PR = 1.271(1.192–1.354); *p* < 0.001, PR = 1.220(1.138–1.308); *p* < 0.001), chronic diseases (multimorbidity: PR = 1.464(1.368–1.566); *p* < 0.001, PR = 1.279(1.181–1.385); *p* < 0.001) and high blood lipids (yes: PR = 1.288(1.228–1.351); *p* < 0.001, PR = 1.130(1.071–1.193); *p* < 0.001) were statistically significant predictors of specialist visits, with the exception of age (60–69 years: PR = 1.097(1.006–1.196); *p* = 0.035 and 70–79 years: PR = 1.195(1.095–1.304); *p* < 0.001) and employment status (inactive: PR = 1.118(1.048–1.193); *p* < 0.001), which showed significant results only in the univariate analysis (Table 3).

**Table 3:** Prevalence ratio analysis: association between sociodemographic characteristics, socioeconomic characteristics, health aspects, and specialist visits.

Variables	Specialist visits			
	Univariable model		Multivariable model	
	PR (95%CI)	*p*	PR (95%CI)	*p*
Sex				
Female	0.992 (0.945–1.041)	0.744	0.971 (0.920–1.025)	0.287
Male	1		1	
Age				
20–29	1.001 (0.760–1.319)	0.992	1.257 (0.920–1.718)	0.151
30–39	0.869 (0.719–1.051)	0.149	0.938 (0.753–1.169)	0.569
40–49	0.997 (0.885–1.123)	0.964	1.137 (0.983–1.315)	0.085
50–59	1.063 (0.967–1.169)	0.208	1.076 (0.956–1.212)	0.226
60–69	1.097 (1.006–1.196)	**0.035**	1.038 (0.943–1.142)	0.446
70–79	1.195 (1.095–1.304)	**<0.001**	1.086 (0.989–1.194)	0.085
80+	1		1	
Region				
Capital	1.051 (0.983–1.124)	0.143	0.986 (0.912–1.065)	0.716
Northern	1.006 (0.941–1.076)	0.860	0.944 (0.879–1.014)	0.116
Central and Western Serbia	0.920 (0.861–0.982)	**0.013**	0.919 (0.859–0.983)	**0.014**
Southern and Eastern Serbia	1		1	
Marital status				
Never married, extramarital union	0.933 (0.826–1.054)	0.264	1.001 (0.872–1.149)	0.991
Divorce, separation, death of a partner	0.953 (0.903–1.006)	0.082	0.955 (0.897–1.016)	0.141
Marriage, extramarital union	1		1	
Education				
Primary	0.800 (0.748–0.856)	**<0.001**	0.755 (0.697–0.818)	**<0.001**
Secondary	0.879 (0.826–0.936)	**<0.001**	0.885 (0.826–0.948)	**0.001**
Advanced	1		1	
Employment status				
Unemployed	0.977 (0.887–1.076)	0.635	0.943 (0.847–1.050)	0.286
Inactive	1.118 (1.048–1.193)	**<0.001**	1.086 (0.988–1.194)	0.087
Employed	1		1	
Well-being index				
Poorest	0.919 (0.870–0.970)	**0.002**	0.933 (0.872–0.997)	**0.042**
Middle class	0.998 (0.937–1.063)	0.955	0.999 (0.933–1.069)	0.969
Wealthiest	1		1	
Self-assessment of general health				
Bad/very bad	1.426 (1.335–1.523)	**<0.001**	1.381 (1.281–1.490)	**<0.001**
Average	1.271 (1.192–1.354)	**<0.001**	1.220 (1.138–1.308)	**<0.001**
Good/very good	1		1	
Chronic diseases				
Multimorbidity	1.464 (1.368–1.566)	**<0.001**	1.279 (1.181–1.385)	**<0.001**
1 disease	1		1	
High blood lipids				
Yes	1.288 (1.228–1.351)	**<0.001**	1.130 (1.071–1.193)	**<0.001**
No	1		1	
BMI				
Malnutrition	0.924 (0.671–1.271)	0.627	0.909 (0.648–1.275)	0.580
Normal	0.927 (0.783–1.099)	0.384	0.963 (0.810–1.145)	0.672
Pre-obesity	0.985 (0.835–1.163)	0.861	1.022 (0.863–1.210)	0.804
Obesity class I	0.985 (0.832–1.166)	0.856	1.027 (0.865–1.219)	0.765
Obesity class II	0.921 (0.762–1.114)	0.398	0.941 (0.776–1.140)	0.533
Obesity class III	1		1	

*Statistically significant results (*p* < 0.05) are presented in bold.

The findings from the univariable prevalence ratio analysis regarding the sociodemographic, socioeconomic and health factors revealed that the key determinants of frequency of hospitalization were all of the factors, except for the region. The respondents who were divorced, had primary education and were unemployed/inactive are 1.2, 1.5 and 1.8/1.9 times more likely to be hospitalized compared to individuals who were married, had advanced education, and were employed. Moreover, poorest participants had 17.7% higher prevalence of hospitalization. With regard to the self-assessment of health, patients who answered bad/very bad (PR = 5.178(4.086–6.561); *p* < 0.001) and average (PR = 2.251(1.755–2.887); *p* < 0.001) were more likely to be hospitalized in comparison to those who defined their health as good/very good. Lastly, in patients with Class II obesity there was a 50.7% reduced probability to be admitted to hospital relative to patients with Class III obesity. The multivariable analysis identified gender, age, self-assessment of general health, chronic diseases and BMI as significant predictors (Table 4).

**Table 4:** Prevalence ratio analysis: association between sociodemographic characteristics, socioeconomic characteristics, health aspects, and hospitalization.

Variables	Hospitalization			
	Univariable model		Multivariable model	
	PR (95%CI)	*p*	PR (95%CI)	*p*
Sex				
Female	0.820 (0.710–0.947)	**0.007**	0.720 (0.605–0.856)	**<0.001**
Male	1		1	
Age				
20–29	0.827 (0.383–1.786)	0.629	2.567 (1.112–5.924)	**0.027**
30–39	0.670 (0.400–1.120)	0.127	1.116 (0.582–2.141)	0.741
40–49	0.455 (0.304–0.682)	**<0.001**	0.903 (0.555–1.468)	0.681
50–59	0.758 (0.584–0.984)	**0.037**	1.095 (0.766–1.566)	0.618
60–69	0.829 (0.660–1.042)	0.108	0.968 (0.732–1.279)	0.817
70–79	1.078 (0.858–1.354)	0.518	1.156 (0.885–1.512)	0.287
80+	1		1	
Region				
Capital	0.996 (0.812–1.222)	0.972	1.037 (0.804–1.337)	0.780
Northern	0.910 (0.741–1.118)	0.368	0.912 (0.724–1.150)	0.437
Central and Western Serbia	0.881 (0.727–1.067)	0.195	0.937 (0.761–1.153)	0.538
Southern and Eastern Serbia	1		1	
Marital Status				
Never married, extramarital union	1.193 (0.860–1.655)	0.291	1.307 (0.906–1.884)	0.152
Divorce, separation, death of a partner	1.186 (1.016–1.385)	**0.030**	1.058 (0.872–1.285)	0.568
Marriage, extramarital union	1		1	
Education				
Primary	1.507 (1.174–1.936)	**0.001**	1.803 (0.804–1.458)	0.601
Secondary	1.266 (0.987–1.624)	0.064	1.313 (0.994–1.735)	0.056
Advanced	1		1	
Employment status				
Unemployed	1.832 (1.356–2.475)	**<0.001**	1.390 (0.988–1.955)	0.059
Inactive	1.962 (1.546–2.490)	**<0.001**	1.389 (0.987–1.956)	0.060
Employed	1		1	
Well-being index				
Poorest	1.177 (0.999–1.387)	**0.050**	0.866 (0.704–1.066)	0.175
Middle class	0.974 (0.791–1.199)	0.803	0.902 (0.721–1.130)	0.371
Wealthiest	1		1	
Self-assessment of general health				
Bad/very bad	5.178 (4.086–6.561)	**<0.001**	4.346 (3.272–5.772)	**<0.001**
Average	2.251 (1.755–2.887)	**<0.001**	1.966 (1.495–2.585)	**<0.001**
Good/very good	1		1	
Chronic diseases				
Multimorbidity	2.929 (2.308–3.716)	**<0.001**	2.018 (1.517–2.685)	**<0.001**
1 disease	1		1	
High blood lipids				
Yes	1.311 (1.121–1.533)	**0.001**	0.967 (0.810–1.154)	0.709
No	1		1	
BMI				
Malnutrition	1.039 (0.108–0.263)	0.926	0.913 (0.401–2.078)	0.829
Normal	0.773 (0.463–2.330)	0.289	0.766 (0.479–1.226)	0.266
Pre-obesity	0.759 (0.481–1.243)	0.245	0.820 (0.519–1.294)	0.394
Obesity class I	0.837 (0.477–1.209)	0.460	0.893 (0.561–1.420)	0.632
Obesity class II	0.493 (0.274–0.888)	**0.019**	0.476 (0.269–0.842)	**0.011**
Obesity class III	1		1	

*Statistically significant results (*p* < 0.05) are presented in bold.

## 4 Discussion

Our study examines how socioeconomic, sociodemographic, and certain health factors may be associated with healthcare utilization among cardiovascular patients.

Primarily, the inclusion of 4,712 individuals in the analysis, which constitutes 36% of the total sample (13,178), suggests that a significant portion of the population may be experiencing cardiovascular problems. Our study emphasized several notable associations between the aforementioned factors. The chi-square test had shown that predominantly, each form of healthcare utilization was most prevalent among the 60–69 age group (general practitioner visits: 33.9%, specialist visits: 33.1%, hospitalization: 31.4%, *p* < 0.001). Self-assessment of general health (bad/very bad) and the presence of multiple chronic conditions were positively associated with general practitioner (PR = 1.037(1.001–1.075); *p* = 0.044, PR = 1.077(1.039–1.117); *p* < 0.001), specialist visits (PR = 1.381(1.281–1.490); *p* < 0.001, PR = 1.279(1.181–1.385); *p* < 0.001) and hospitalization (PR = 4.346(3.272–5.772); *p* < 0.001, PR = 2.018(1.517–2.685); *p* < 0.001).

Additionally, the relatively high prevalence of cardiovascular issues could reflect an accurate representation of the health status of the population, which could further suggest that the survey effectively captured individuals with varying degrees of cardiovascular disease and this further underscores the additional burden that these diseases impose on the healthcare system and its utilization, which is offering a comprehensive overview of public health in Serbia. It also helps in shaping national health strategies aimed at reducing the burden of cardiovascular diseases on the healthcare system.

According to the study in Croatia, there is a constant correlation between socioeconomic and sociodemographic factors and healthcare utilization among patients with chronic illnesses, particularly those with cardiovascular diseases (23). A previous study has shown that lower socioeconomic status has been linked to reduced utilization of other preventive care services (24). Our study did manage to establish some notable variations across various socioeconomic groups and educational attainment levels in practically every facet of healthcare utilization. People with a lower socioeconomic standing—indicated by a secondary education and low income, as well as those with an inactive employment status had utilized more healthcare than those with greater educational and income levels. Our findings are in line with another study, which showed that cardiovascular diseases continue to place a heavy load on healthcare. The study’s population (adults ≥40 years of age) included 1872 cardiovascular patients, out of whom older (≥75, 31.9%) patients, males (51.9%), people belonging to middle income layer (44.8%) and with secondary level of education (32.9%) had been more prone to healthcare utilization (25).

Geographically, the prevalence of cardiovascular healthcare utilization varies, and individuals who live in regions where managing their medical condition is challenging tend to fare poorly. A major contributing reason to the growing health disparity between urban and rural communities and subsequent differences in cardiovascular risk factors is access to healthcare. Urban residents have more access to healthcare services than those in rural areas, which lowers morbidity and exacerbates regional health disparities (26). A cross-sectional study from the 2013 National Health Survey in the Republic of Serbia among senior cardiovascular patients found that the majority who lived in the country’s eastern and southern region had more general practitioners visits. As a consequence of lower density of specialty care in this area, patients were compelled to frequently visit the primary healthcare institutions. Accordingly, specialist visits were more common among the population that resided in the capital region and had a higher income and educational level, which is not in accordance with the results we found (Central and Western region, secondary education and the lowest layer when it comes to income) (27).

Researches in Ireland in 2019 and 2024 demonstrated the inequality in healthcare utilization, the enormous impact that chronic non-communicable diseases, particularly cardiovascular disease have on general practice. It pointed that the cardiovascular healthcare usage was mainly managed by general practitioners. Cardiovascular patients were predominantly treated in primary healthcare, which supports the findings of our research (85.9%, *p* < 0.001). This is consistent with the chronic character of cardiovascular diseases, whereas acute treatment is frequently needed for exacerbations and primary care is expected to handle the majority of the disease’s burden (28, 29). Accordingly, the aforementioned German study discovered that in the last year, general practitioners were visited by patients in the following percentage: 33.2% (*p* < 0.001) by men and 35.1% (*p* < 0.001) by women, specialists were visited by a smaller percentage (15.2%, *p* < 0.001 and 16.8%, *p* < 0.001), while the number of hospitalized cardiovascular patients was 38% (*p* < 0.001) and 38.1% (*p* < 0.001) (30). A research in older people with cardiovascular disease in China concluded that during period of the 12 months, 17.7% of patients required hospital care (comparable findings with ours – 13.6%), while over half (54.9%) of the patients stated that they had received outpatient care, whereas our result—85.9%, was substantially higher (31).

The identification of healthcare-related factors associated with inadequate hypertension control in Serbia showed a statistically significant difference in the utilization of the services of general practitioners and specialists in relation to age (>66 years old—49.4%, 60.2%, *p* = 0.015) and education status (primary—47.6%, 61%, *p* = 0.004) (32), while in our case, most patients visiting general practitioners and specialists had a secondary level of education. The aforementioned study also showed that outpatient care varied by household wealth level (pro affluent patients) and it was more frequently used as the patients grew older (31), which is entirely consistent with our study, except for the 80 + age group.

Self-rated health has a substantial correlation with cardiovascular morbidity and is utilized extensively as a health indicator. The evaluation of one’s own health has grown in significance over the past few decades and is frequently employed in healthcare surveillance (33). In the study on inequalities in the utilization of health services in Serbia, which was also part of the National Health Survey for the population of Serbia in 2006, it was determined that general practitioner visits were more frequent among those who rated their general health as poor. Hospitalization was also negatively correlated with an individual’s perception of their own health, which is in accordance with our study (7). According to research conducted in China among older adults patients with chronic disease, there was significantly lower result in self-rated health in the hypertension group compared to the other chronic illness groups (34).

In the study of older US individuals an optimal diet and maintaining a normal body mass index (BMI) were correlated with a decreased risk of cardiovascular disease-related inpatient and outpatient visits (35). The UK study in the European Journal of Preventive Cardiology showed similar results and stated that patients with diagnosed cardiovascular illnesses had higher numbers of general practitioner visits among all BMI categories. Surprisingly, compared to patients with pre-obesity or obesity class I and II, individuals with healthy weight had a higher frequency of hospitalizations amongst patients with diagnosed cardiovascular illnesses, which is not supported by our data (pro- pre-obesity) (36). When we investigated the issue of another health factor, high blood lipids, we concluded that participants with high blood lipids exhibited a negative correlation with general practitioner and specialist visits among cardiovascular patients. This finding contrasts with the results of a study conducted in Croatia (23).

In summary, the analysis identified several significant predictors of cardiovascular healthcare utilization, emphasizing distinct and overlapping factors that impact various types of healthcare use. This justifies the necessity for additional research on the factors influencing cardiovascular patients’ healthcare utilization. By identifying them, strategies that effectively prevent complications and lessen cardiovascular burden can be developed, which can have a substantial impact on the expenses associated with prevention, treatment and rehabilitation. Unfortunately, the number of cardiovascular patients in need of healthcare is more than the capacity of the current healthcare system and the system might not be able to handle the increase in older adult patients. Nonetheless, efforts must be made to provide financial resources to assist people and the healthcare system. The best ways to do this are through data-driven policy and institutional changes, as well as interventions aimed at raising the socioeconomic standing of cardiovascular patients. The broader policy and public implications for cardiovascular healthcare utilization revolve around ensuring equitable access to care, optimizing resource allocation, and addressing disparities in health outcomes. Policies should focus on improving healthcare infrastructure, especially in underserved areas, and promoting preventive measures to reduce the burden of cardiovascular diseases. Additionally, addressing social determinants of health, such as income and education, can play a crucial role in reducing the utilization gap and improving health equity.

### 4.1 Limitations

Certain limitations must be acknowledged. This research may be subject to selection bias, as certain groups (e.g., living in isolated rural areas or those who are institutionalized) might be underrepresented. Reporting bias may arise from misreporting of cardiovascular healthcare visits due to recall errors or social desirability. To minimize bias in this research, random sampling was used to ensure a representative population. Standardized data collection procedures were applied to maintain objectivity and reduce measurement errors. Trained interviewers followed strict protocols to prevent interviewer bias, while validated questionnaires minimized reporting bias. Additionally, statistical weighting was employed to correct for non-response bias, ensuring more accurate and generalizable findings. Furthermore, as a cross-sectional study, this research can reveal associations between sociodemographic, socioeconomic, health factors and cardiovascular healthcare, but cannot determine causal relationships. To better understand how healthcare utilization change over time, longitudinal studies would be more suitable.

## 5 Conclusion

In conclusion, sociodemographic, socioeconomic factors and some aspects of health status have a significant impact on the cardiovascular healthcare, thereby precise recommendations for facilitating appropriate healthcare utilization should be established. A top priority for academics, physicians and public health specialists is to keep emphasizing the significance of sociodemographic and socioeconomic determinants in lowering cardiovascular complications, as well as strengthening cardiovascular healthcare.

## Data Availability

The raw data supporting the conclusions of this article will be made available by the authors, without undue reservation.
