# Supplementary material for: The effect of sociodemographic, socioeconomic, and health factors on healthcare utilization in cardiovascular patients in Serbia: a part of National Health Survey
Source: Front Public Health. 2025 Jul 17;13:1569741. doi: 10.3389/fpubh.2025.1569741 (PMC12312653; doi:10.3389/fpubh.2025.1569741)

Supplementary data 1. Patients with Cardiovascular Diseases in Percentages

Study population N (%)	Cardiovascular Disease
4217 (89.5%)	Hypertension
1293 (27.4%)	Angina Pectoris
201 (4.3%)	Myocardial Infarction
149 (3.2%)	Stroke

Supplementary data 2. Exclusion Flowchart of the Participants

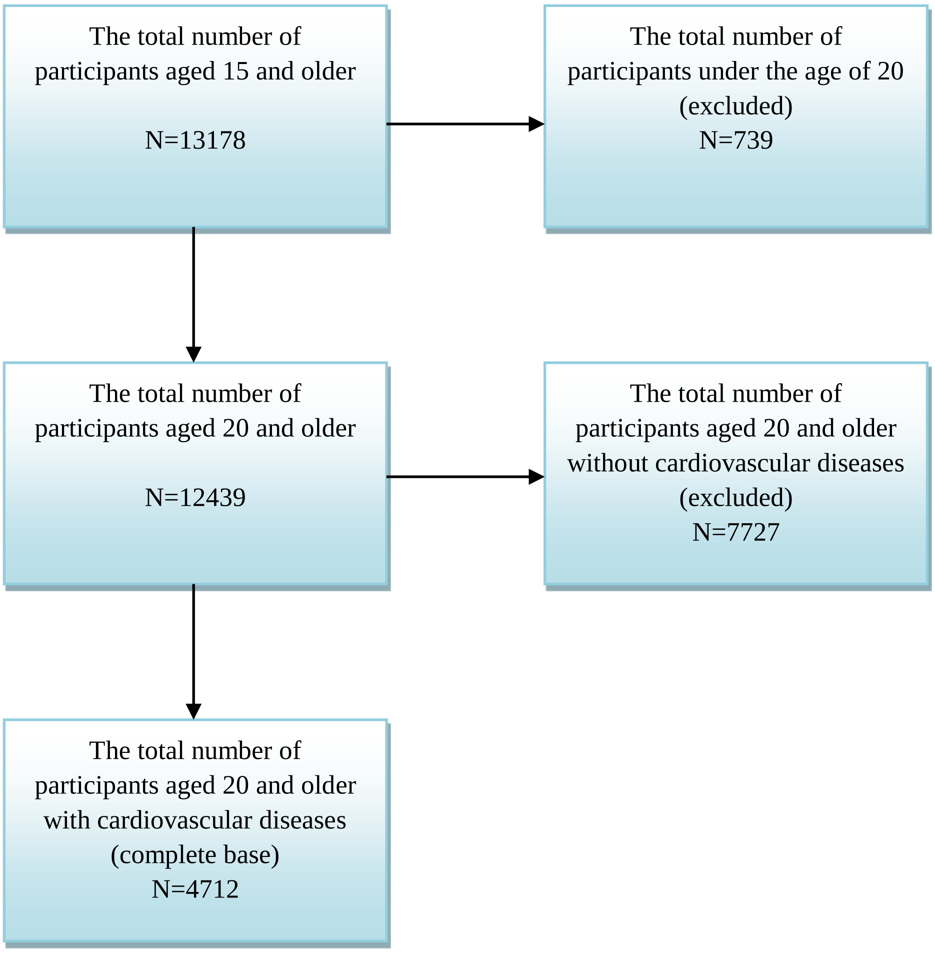

Supplement: Supplementary file 1 [file Data_Sheet_1.pdf]
